# A Case of Supernumerary Incisors in a Young Male Child: A Rare Occurrence of Four Central Incisors in the Maxillary Anterior Region

**DOI:** 10.7759/cureus.35047

**Published:** 2023-02-16

**Authors:** Jeni Ann Mathew, Ranjit Kamble, Simran Das, Sumukh Nerurkar

**Affiliations:** 1 Department of Orthodontics, Sharad Pawar Dental College, Datta Meghe Institute of Higher Education and Research, Wardha, IND; 2 Department of Pediatric and Preventive Dentistry, Sharad Pawar Dental College, Datta Meghe Institute of Higher Education and Research, Wardha, IND

**Keywords:** transitional occlusion, supernumerary teeth, morphology, anterior maxilla, central incisor

## Abstract

Multiple supernumerary teeth in the central incisor region are an uncommon occurrence; the most frequently occurring type of supernumerary teeth are mesiodens. A 10-year-old male had reported to Sharad Pawar Dental College with the chief complaint of extra teeth. An intraoral examination revealed the presence of two labially placed incisors and two palatally placed incisors at a transitional phase of dentition. During a radiographic examination, the maxillary occlusal view revealed four incisors with similar morphology. Extraction of the palatally placed incisor was done under local anaesthesia which was followed by alignment of the anterior teeth and closure of space. Supernumeraries that have erupted should always be removed, unless the teeth next to them are absent, in which case they should be left in place. After alignment, closure of spaces was done since the permanent canines had not yet erupted; it was decided against trying to completely close any gaps between the teeth.

## Introduction

The thirtieth week of pregnancy marks the beginning of the development of the central incisor in the maxilla. Beginning three to four months after childbirth, calcification progresses until it reaches the crown by the age of five. After the first permanent molars, it is frequently seen that incisors erupt [1]. Supernumerary teeth are extra teeth present in primary and permanent dentition. Mesiodens occur in between the central incisors and appear more frequently than other supernumerary teeth, usually occurring one or more in number in the maxilla or mandible, unilaterally present or bilateral [2].

This occurrence has been found to be 1.5-3.5% in subjects with permanent dentition and 0.3%-0.8% in children having deciduous dentition [3]. In the case of mesiodens in the maxillary midline, they can be single or multiple in number. Mesiodens can hinder normal eruption pathways and deviate into spaces in the maxilla like the nasal cavity or maxillary sinus. They can be present in the labial side or palatal side of the arch as erupted or impacted [4]. Early treatment of malocclusion is preferred by many clinicians as they have short-term and long-term benefits of achievement of occlusal and functional harmony. The pedodontist has a crucial role in the early detection and intervention of malocclusion. The chance of a long span of orthodontic treatment can be prevented [5]. The present case describes a rare case of a 10-year-old male child with four maxillary incisors with similar crowns and roots.

## Case presentation

**Figure 1:**
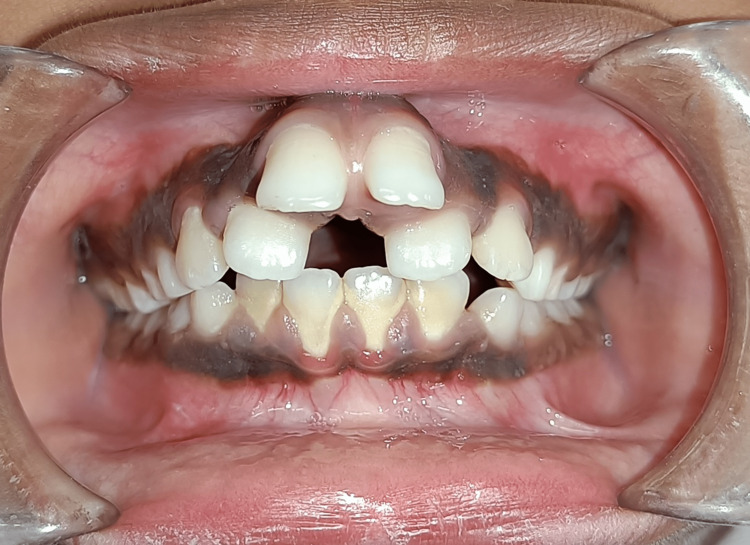
Pretreatment frontal view

**Figure 2:**
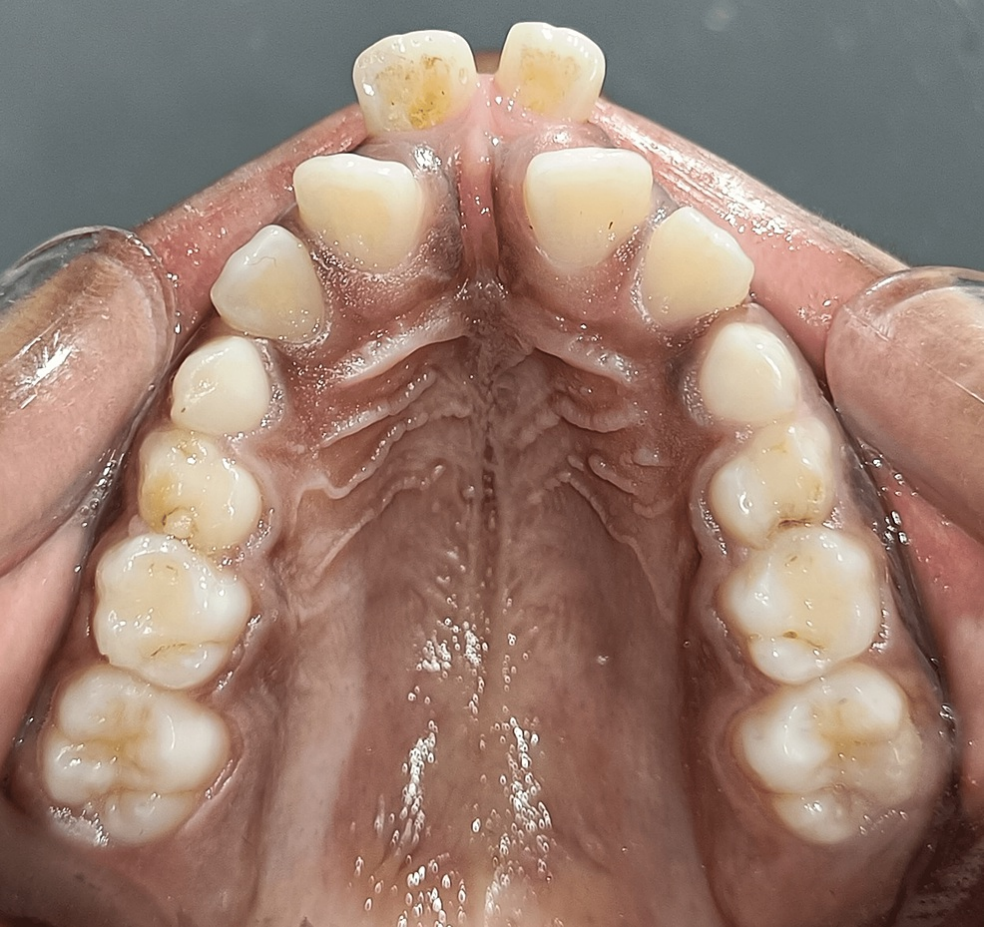
Pretreatment occlusal view

**Figure 3:**
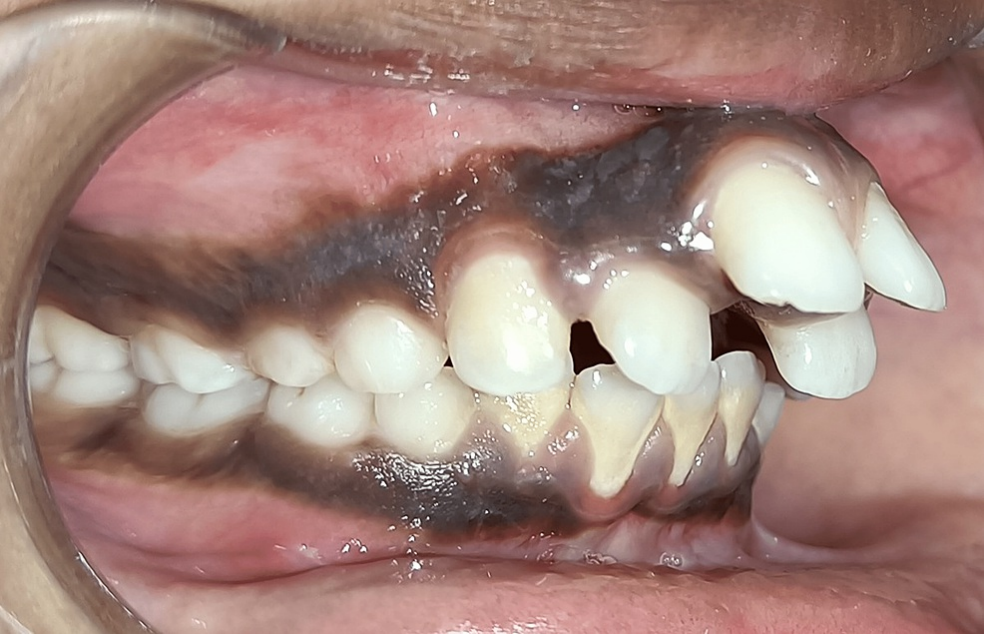
Pretreatment right lateral view

**Figure 4:**
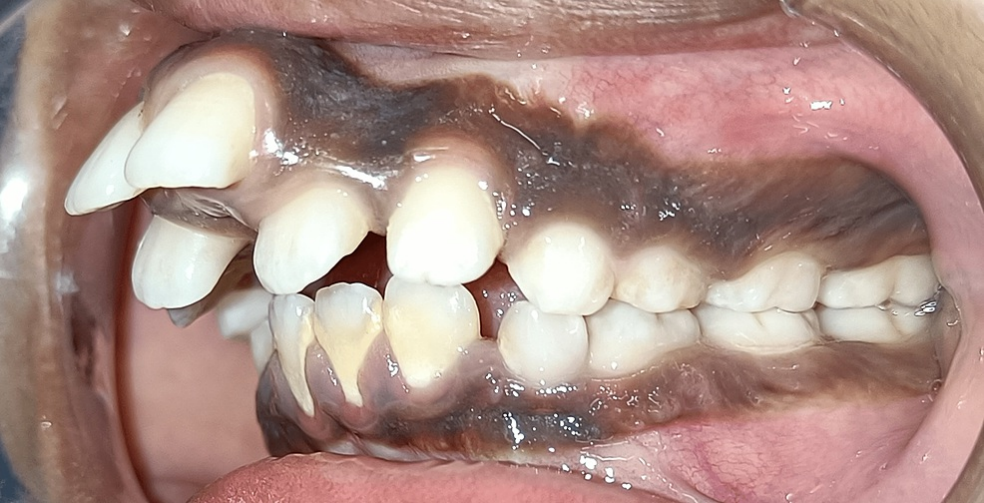
Pretreatment left lateral view

**Figure 5:**
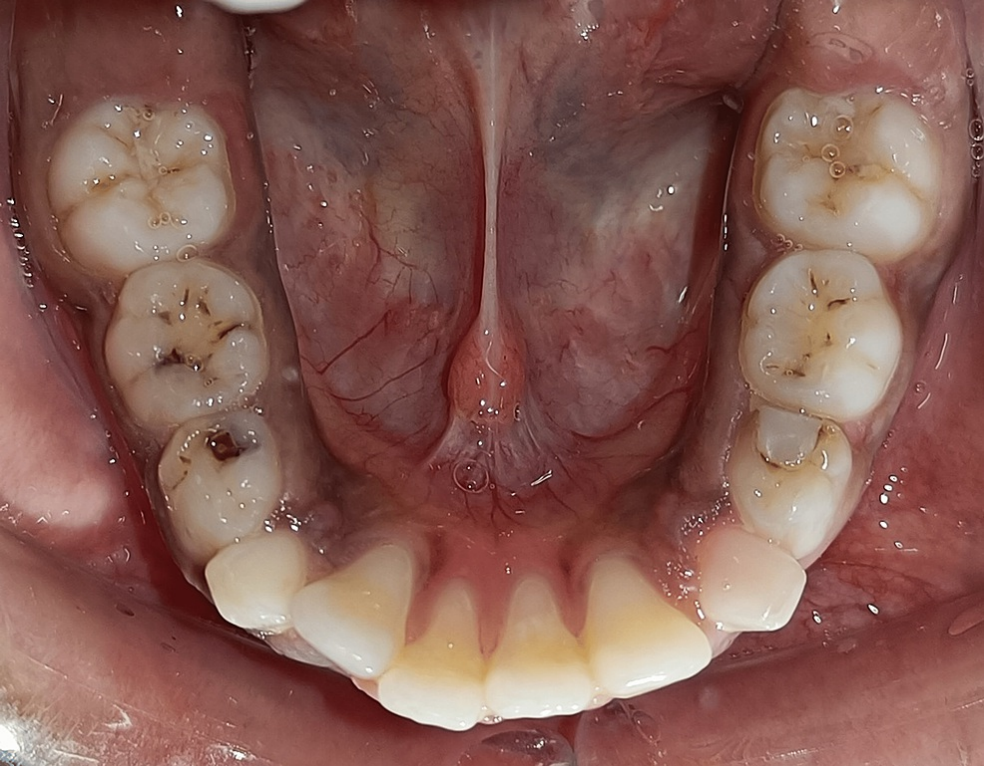
Pretreatment mandibular occlusal view

**Figure 6:**
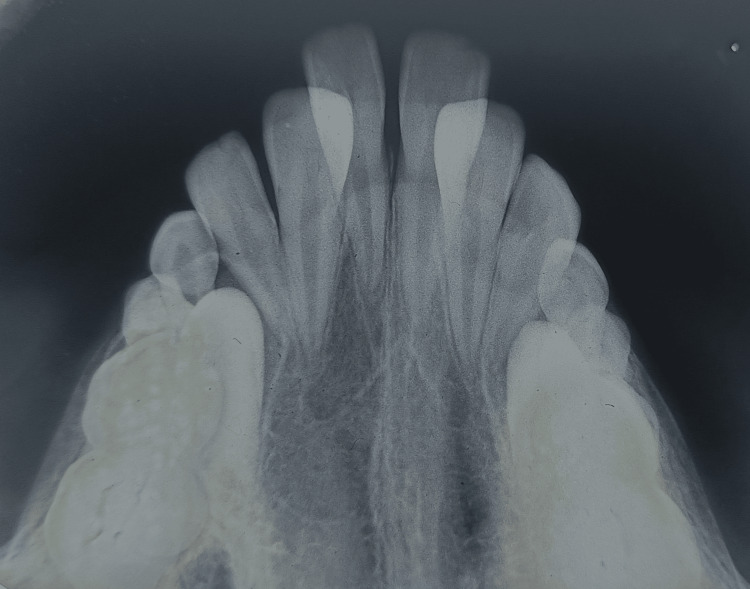
Pretreatment occlusal radiograph

A 10-year-old male child had reported to the Sharad Pawar Dental College with the chief complaint of poor aesthetics. The parents complained about the reluctance of the child to attend school as he was ridiculed for his extra teeth. Family history was noncontributory and syndromic conditions were also ruled out. Medical history was also noncontributory. Transitional occlusion was seen in the child. The presence of four incisors was seen. Two incisors were present labially and two were present palatally, succedaneous lateral incisor was seen with deciduous canine, the first and second molar, and succedaneous first molar (Figures 1-5). The maxillary occlusal radiograph revealed that all four incisors had similar crown and root morphology (Figure 6).

**Figure 7:**
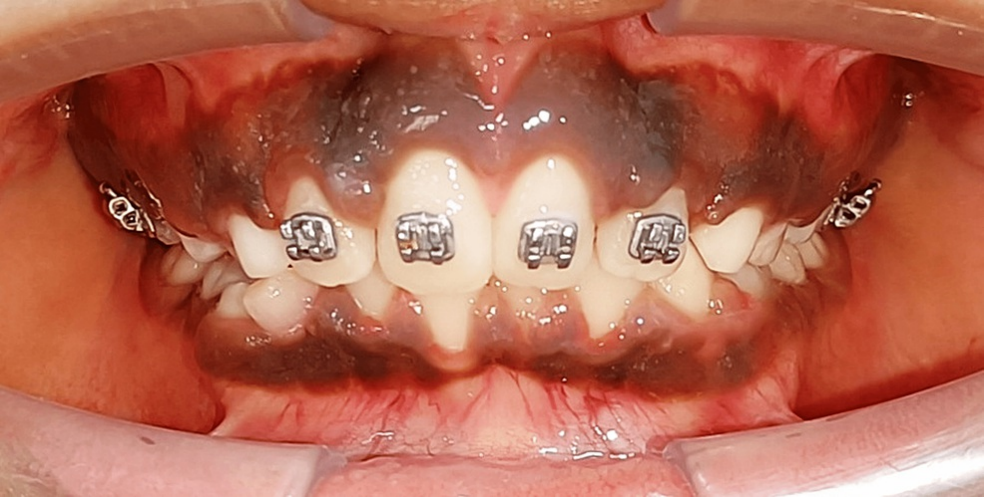
Post-treatment frontal view

**Figure 8:**
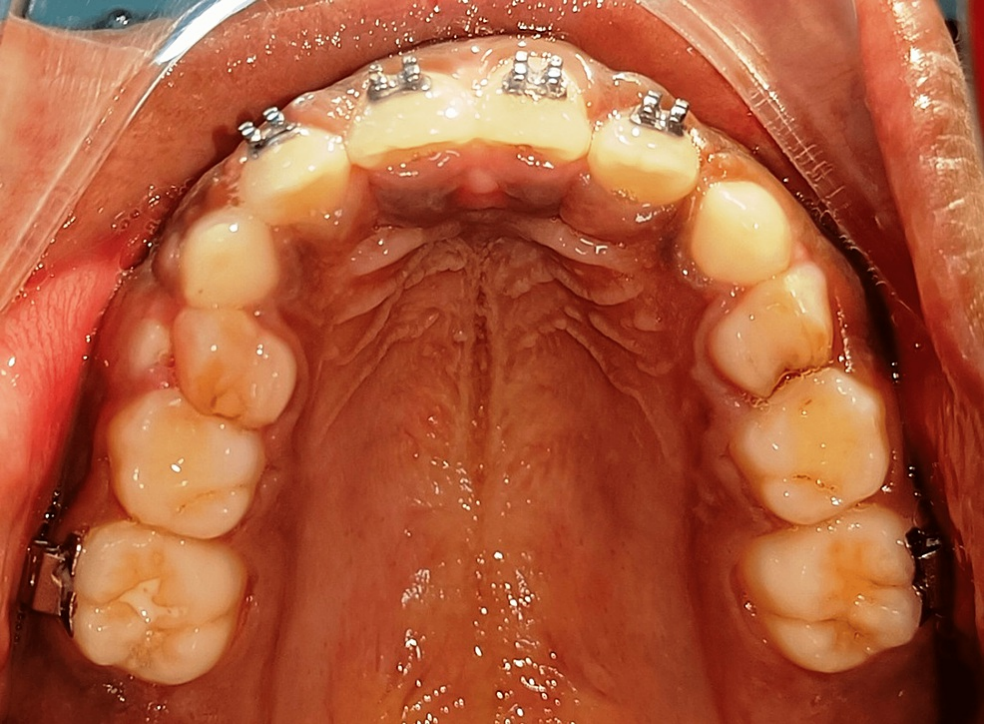
Post-treatment maxillary occlusal view

**Figure 9:**
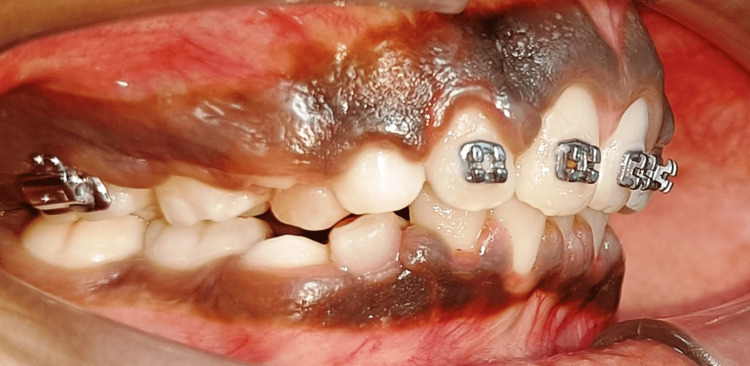
Post-treatment right lateral view

**Figure 10:**
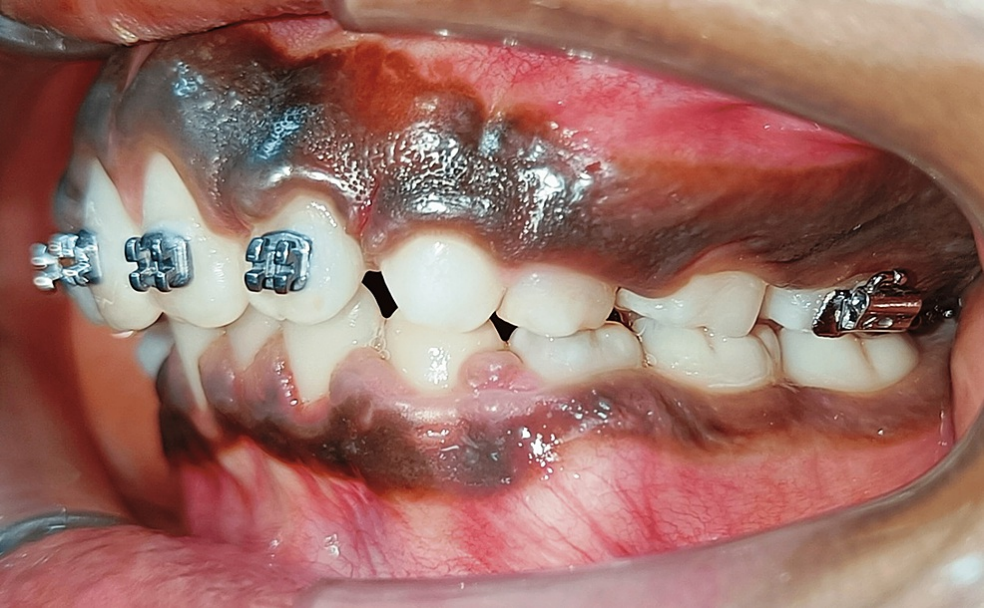
Post-treatment left lateral view

**Figure 11:**
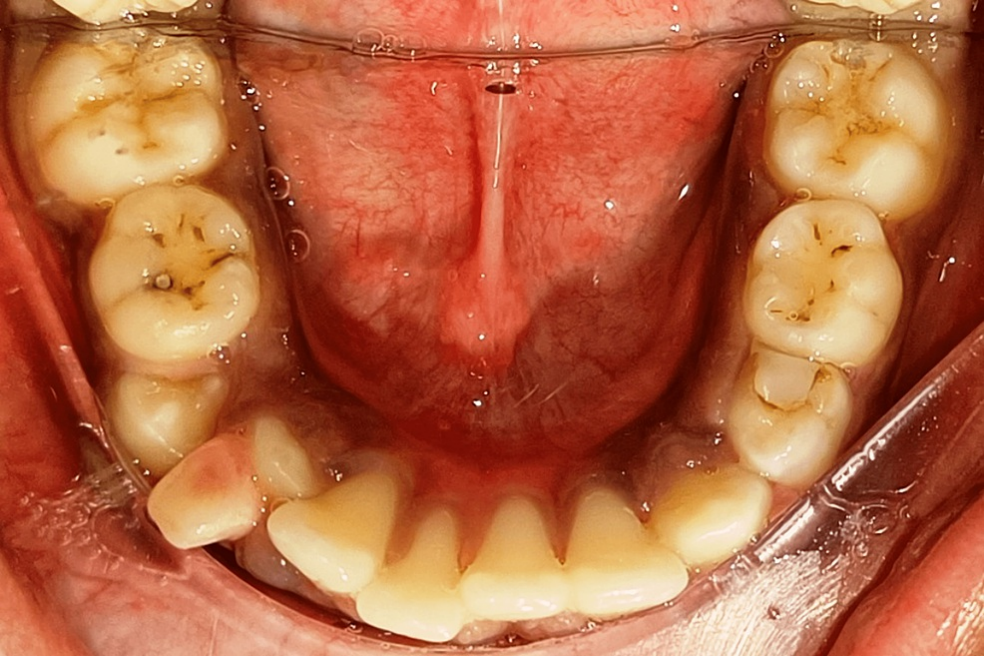
Post-treatment mandibular occlusal view

The treatment objective was to treat the un-aesthetic smile of the child. Two possible treatment plans were formulated. Plan 1 formulated included extraction of labially placed incisors and shaving of labial bone and Plan 2 included extraction of palatally placed incisors and lip exercises with the objective of post-extraction of incisor and partial closure of space. The patient had agreed to undergo Plan 2. Extraction of palatally placed incisors under local anaesthesia was done atraumatically followed by orthodontic intervention. The fixed appliance system used in this case was the McLaughlin, Bennett, Trevisi (MBT) system with a 0.022 slot. The orthodontic intervention involved alignment by a progression of wire with 0.016 nickel-titanium (NiTi) to 0.019 × 0.025 stainless steel (SS) and closure of space partially with 0.019 × 0.025 SS as permanent canine was yet to erupt (Figure 7-11). 

At the start of treatment, the patient had an indifferent attitude towards treatment. During the extraction procedure, the patient was apprehensive. The treatment in this patient not only involved atraumatic extraction by a pedodontist and orthodontic intervention by an orthodontist but also repetitive counseling in every appointment to build the morale of the child. After addressing the chief complaint of the patient, the shedding of deciduous teeth and eruption of succedaneous teeth was scheduled to be monitored over the next 10 months. Final finishing and detailing of occlusion were planned for the patient. 

## Discussion

Supernumerary teeth have an origin that is not fully known. Theories explaining the occurrence of supernumerary teeth are recorded in literature. The atavism hypothesis postulates that certain features could reappear during the evolutionary shift. On the other hand, the dichotomy theory postulated that the split in tooth germ could be symmetrical or asymmetrical, resulting in extra teeth varying in size or morphology or both. The researchers are in support of dental lamina hyperactivity which postulates that the dental lamina exhibited localized, independent, and conditioned activity [6].

The etiology of these conditions is unclear. Many investigators have postulated causes of supernumerary teeth like embryological, development dysplasia, etc. According to Syriac et al., 93.3% of the supernumerary teeth were found in the maxilla of the anterior region [7]. De Oliveria Gomes et al. stated that the maxilla is the site where 91.3% of supernumerary teeth have been reported [8].

Kim et al. reported the occurrence of single extra teeth at 69.2% [9]. Asaumi et al. found the occurrence of three mesiodens at 1% [10]. The occurrence of four supernumerary teeth in the anterior maxilla is rare [11]. The complication created by supernumerary teeth is the ectopic eruption of an adjacent tooth, overcrowding, spacing issues, or the development of follicular cysts if unerupted as has been quoted in the literature. Mesiodens were extracted in 62% of instances, followed by orthodontic intervention. Supernumeraries can be extracted when they have erupted completely and retention of teeth is recommended when teeth adjacent to it are missing [8,12-14].

Shah et al. and Amaralal et al. recommended that supernumerary teeth can be left in the arch if they are asymptomatic [15,16]. Omer et al. recommended extraction when the tooth would be at Demirijian stage C; the removal of teeth would show few difficulties [17]. Mason et al. and Arangannal et al. recommended extraction immediately post early identification [3,18]. But in the current case, the patient wanted to remove the supernumerary teeth.

In the plans formulated for the child, Treatment Plan 1 formulated included extraction of labially placed incisors. The main concern of the patient was aesthetics. Extraction of labially placed teeth would address convexity of the face with results achieved early. But the procedure involved shaving of labial bone. Treatment Plan 2 included extraction of palatally placed incisors and lip exercises to remodel the bone. Amongst these two plans, the patient opted for a less invasive procedure. Post-extraction closure of space was aimed partially as the canine was yet to erupt. The change in the appearance of the child improved acceptance among peers and thereby the confidence of the child. This case not only shows the treatment of a rare occurrence but reestablishes the already proven significance of optimum muscle exercise in various orthodontic interventions.

## Conclusions

Treatment of supernumerary teeth not only involves the mechanical and biomechanical aspects of treatment but also counseling of the child in every appointment as the child was affected greatly by peer pressure and constant ridicule in their social circle. Treatment involved extraction of the palatally placed incisor followed by orthodontic intervention. Complete closure of spaces was not done as the canines were yet to erupt and had not reached occlusion.
